# Association between +45T>G adiponectin polymorphism gene and type 2 diabetes mellitus and metabolic syndrome in a Venezuelan population

**DOI:** 10.12688/f1000research.16890.1

**Published:** 2019-03-14

**Authors:** María Patricia Sánchez, Carem Prieto, Endrina Mujica, Kendry Vergara, Enifer Valencia, Eudymar Villalobos, Mayerlim Medina, Michael Parra, Rosanna D'Addosio, Kyle Hoedebecke, Johel E. Rodríguez, Valmore Bermudez

**Affiliations:** 1 Centro de Investigaciones Endocrino-Metabólicas “Dr. Félix Gómez”, Facultad de Medicina, Universidad del Zulia, Maracaibo, Venezuela; 2 Escuela de Bioanálisis, Facultad de Medicina, Universidad del Zulia, Maracaibo, Venezuela; 3 Carrera de Medicina, Unidad Académica de Salud y Bienestar, Universidad Católica de Cuenca, Cuenca, Ecuador; 4 Escuela de Medicina, Facultad de Medicina, Universidad del Zulia, Maracaibo, Venezuela; 5 Departmento de Salud Pública, Escuela de Medicina, Universidad del Zulia, Maracaibo, Venezuela; 6 Department of Family Medicine, Uniformed Services University of the Health Sciences, APO, Armed Forces Pacific, 96205, USA; 7 Department of Family Medicine, WONCA Polaris, APO, AP, 9605, USA; 8 Facultad de Ingenierías, Universidad Simón Bolívar, Cúcuta, Colombia; 9 Universidad Simón Bolívar, Facultad de Ciencias de la Salud, Barranquilla, Colombia

**Keywords:** metabolic syndrome, type 2 diabetes mellitus, ADIPOQ gene, polymorphism, DNA

## Abstract

**Background:** Adiponectin (ADIPOQ) is a hormone primarily synthesized by adipocytes and encoded by the *ADIPOQ* gene, which exerts anti-inflammatory, antiatheratogenic and insulin sensitizing functions. It has been shown that its plasma concentrations are decreased in individuals with metabolic syndrome (MS) and type 2 diabetes mellitus (DM2), which could be due to variations in the gene coding for this protein. The aim of this study was to detect the +45 T>G polymorphism of the *ADIPOQ* gene in subjects with DM2 and MS in Maracaibo municipality, Zulia state, Venezuela.

**Methods:** A total of 90 subjects who attended the Center for Metabolic Endocrine Research "Dr. Félix Gómez" were enrolled for this study, 46 of which had MS-DM2 and 44 of which were healthy control individuals. Genomic DNA was extracted from blood samples and PCR-restriction fragment length polymorphism analysis was carried out for the promoter region of the *ADIPOQ* gene. Likewise, the +45 T> G polymorphism was identified and correlated with MS and DM2 in the studied population.

**Results:** The most frequent allele in both groups was the T allele, and the predominant genotype was homozygous T/T (79%). Genotypes with heterozygous T/G and G/G homozygous polymorphism were more frequent in the control group than in the MS-DM2 group. Regarding the individuals with T/G and G/G genotypes, statistically significant lower mean values ​​were found for fasting glucose, total cholesterol, triacylglycerides, abdominal circumference, and for the medians of systolic and diastolic blood pressure. Odds ratio were calculated for the presence or absence of MS and DM2.

**Conclusions:** The results suggested that the presence of the G allele exerts a protective effect on the carrier individuals, thus avoiding the appearance of the aforementioned metabolic alterations.

## Introduction

Adiponectin contains 244 amino acids ^1^ and this protein is expressed almost exclusively in white adipose tissues where it has anti-diabetic, anti-inflammatory, and anti-atherogenic properties in addition to cardioprotective effects ^2, 3^. It is considered one of the most abundantly secreted adipokines by the adipocyte, because its plasma concentrations in humans vary between 5–30 μg/ml, and although this values vary according to sex (generally higher in women) ^4^, adiponectin represents 0.01% of total plasma proteins ^3^. Unlike other known adipokines, its levels are decreased in insulin-resistance associated states, such as obesity, type 2 diabetes mellitus (DM2) and metabolic syndrome ^5^.

Metabolic syndrome (MS) is a complex of interrelated risk factors for the development of cardiovascular diseases and diabetes mellitus (DM) ^6^. The prevalence of MS in the world is very high; 45% of the population over 50 years old and about 20% of the population under 50 years of age have MS ^7^. Patients with MS have twice the risk of developing cardiovascular disease in 5 to 10 years after being diagnosed ^8^, increasing the risk of myocardial infarction ^9^.

Epidemiological studies conducted in the U.S have shown that the general prevalence of MS is 24% in Caucasian populations, which increases according to age to more than 30% in people over 50 years of age and more than 40% in people over 60 years of age ^10^. In a study carried out in Maracaibo, Venezuela, the prevalence of MS was 42.7%, presenting men at a higher risk when compared with women; as age increases, the risk also increases progressively. Similarly, diabetic individuals had up to 4 times higher risk of suffering from MS ^11^.

Diabetes mellitus is one of the main causes of morbidity and mortality, as well as an important cardiovascular risk factor and a poor prognosis in patients with established cardiovascular disease ^12^. Its prevalence has drastically increased in the last 20 years and it is estimated that by the year 2030 the number of affected will exceed approximately 400 million people ^12^, which translates into a 54% increase ^13^.

Some 422 million people suffer from diabetes in the world, totaling one out of every 11 people. It is estimated that in 2012, 1.5 million people died as a direct consequence of diabetes. According to WHO projections, diabetes will be the seventh greatest cause of mortality in 2030 ^14^. In Venezuela, according to the 2011 Ministry of Public Power of Health, diabetes mellitus represents the fourth-leading cause of death. This data corresponds with WHO data. Furthermore, diabetes mortality projections remain high over the foreseeable future ^15^. In an analysis conducted on a Venezuelan population (Maracaibo City, Zulia State) about the DM2 prevalence, it was found that 8.4% of the evaluated population had the disease, which is similar to that shown in our continent, in the Carmela study, which took in consideration seven cities in Latin America, evaluating more than 11,000 individuals aged between 25 and 64 years; this study reported a general prevalence of 7%, where the one shown in Mexico City stands out (8.9%) since is higher ^12^.

The serum concentration of adiponectin is reduced in individuals with obesity, DM2, insulin resistance, obesity, dyslipidemia and coronary disease ^16, 17^. With an link to lower lipid oxidation, higher triglyceride content, and reduced levels of insulin-dependent signaling—reduced concentrations of adiponectin have been noted in the pathophysiology MS and DM2 ^18^.

The *ADIPOQ* gene is located on chromosome 3q27, consists of 3 exons and is 15.8 kb long ^1^. Genetic variants in *ADIPOQ* can lead to substantial changes in adiponectin levels, which provides convincing evidence that subtle alterations in the genetic code can modify the levels of this hormone, large enough to significantly affect the health, as mentioned before ^1^. Several studies have determined that different polymorphisms (SNPs) have been located in the adiponectin gene, which can affect both the transcription and the activity of this hormone. Among the most prominent are the polymorphisms +45 T>G, +276 G>T, -11.377 C>G and -11.391 G>A ^19^.

Studies carried out in different populations, associated the +45T>G polymorphism of the *ADIPOQ* gene with the development of DM2, insulin resistance, obesity, coronary artery disease and hypoadiponectinemia ^19^. Other studies reported that subjects with the T/G or G/G genotype at position 45 have a higher risk of developing DM2 when compared to those with T/T genotype ^20^. Likewise, it has been observed that subjects who present the G allele of the +45T>G polymorphism have lower plasma levels of adiponectin ^19^.

Due to such significant findings, this study aimed to determine nucleotide alterations of the *ADIPOQ* gene, especially the +45T>G polymorphism and its relationship with DM2 and MS, in adult individuals of the Maracaibo municipality of Venezuela. The study aimed to identify cases of people carrying the alteration indicated in this gene, which could be related to DM2 and MS. In this way, the presence of the polymorphism would serve as a predictor for the development of MS and/or DM2 in the future ^16, 21^, guaranteeing the possibility that any person with a predisposing genotype can take opportune measures to prevent these diseases.

## Methods

### Subjects

A total of 90 adult individuals, of which 46 had MS-DM2 and 44 were healthy controls, were enrolled in this study, which was carried out from January 2015 to January 2016. Participants were recruited through a mixed strategy. Some individuals were contacted by phone while in remote locations we made contact with the community leaders in order to verify the size and organization of each neighborhood since public records prove inaccurate. After this, we proceeded to draw sectors and blocks, and then, together with the community leaders, contact the members of the selected houses directly. These individuals participated in the Metabolic Syndrome Prevalence Project ^11^ of the Metabolic Endocrine Research Center "Dr. Félix Gómez" (CIEM) of the Faculty of Medicine of University of Zulia performed from 2007 to 2008. The present study size was the same as the previous one. The participants signed an informed consent form, where they were explained everything related to the project and the handling of their samples and tests. This research was approved by the CIEM bioethics committee and complies with the Helsinki declaration. The studied subjects were chosen since they met the predetermined inclusion criteria and with the absence of selected exclusion criteria, depicted in Table 1. In order to make the diagnosis of MS and DM2, 2009 Consensus ^6^ and ADA ^22^ criteria was used respectively.

**Table 1.** Eligibility criteria for each studied group.

	Healthy controls group	DM2+ MS group
Age	>18 years old	>18 years old
WC	<98.15 cm males and < 91. 50 cm females ^a^	≥98.15 cm males and ≥91.50 cm females ^a^
SBP	<130 mmHg	≥130 mmHg
DBP	<85 mmHg	≥85 mmHg
HDLc	≥40 mg/dl males; ≥50 mg/dl females	<40 mg/dl males <50 mg/dl females
FG	< 126 mg/dl	≥ 126 mg/dl
TG	<150 mg/dl	≥150 mg/dl
Diagnosis of DM2+ MS	Absent	Present
Pharmacologic treatment for DM2 and/ or metabolic conditions	Absent	Present

^a^ Regarding Maracaibo, Venezuela these values are adjusted to the population ^11^. WC, Waist circumference; SBP, Systolic Blood Pressure; DBP, Diastolic Blood Pressure; HDLc, Cholesterol bound to high density lipoproteins; FG, fasting glucose; TG, triacylglycerides; DM2, type 2 diabetes mellitus; MS, metabolic syndrome.

### Anthropometry, body composition and biochemistry analysis

The following variables were measured in the subjects:

1. Waist circumference: taken with a graduated measure tape in centimeters at an equidistant point between the costal margin and the anterior superior iliac spine.
2. Body mass index: estimated by dividing the kilograms of weight by the square of the height in square meters (BMI = kg/m ^2^). A TANITA electronic scale was used to obtain the body weight. To measure the size, a height rod was used.
3. Blood pressure: measured by the auscultatory method, for which a calibrated and properly validated sphygmomanometer was used.
4. Levels of fasting glucose, total cholesterol, triacylglycerides and HDLc (cholesterol bound to high density lipoproteins): (previous fasting from 8 to 12 hours), for which 5 cm ^3^ of blood was drawn from each individual obtained by antecubital venipuncture, which was placed in test tubes and centrifuged to 4000 rpm for 10 minutes. After this, the serum was extracted and placed in polypropylene test tubes for subsequent freezing at -70°C. The time between taking the sample and processing did not exceed three months. To determine the fasting glucose, an enzymatic-glucose-colorimetric enzyme kit (Glucose Liquicolor Ref. 10260300, Human Diagnostics) was used. For the quantification of total cholesterol, triacylglycerides and HDLc, commercial enzyme-colorimetric kits (Cholesterol Liquicolor Ref. 10028300; Triacylglicerides liquicolor mono Ref. 10724300; HDL Cholesterol liquicolor Ref. 10084300; all from Human Diagnostics) were used.
5. Low-density lipoprotein bound to cholesterol (LDLc), calculated by the Friedelwald formula: (LDL-c = total cholesterol - (VLDL=c + HDL-c), where VLDL-c = triacylglycerides/5 (mg/dl) (provided that the triacylglycerides cannot exceed 400 mg/dl).
6. Ultrasensitive CRP (usCRP): Turbidimetric method with latex for the quantitative determination of C Reactive Protein (CRP hs Turbitest AA Ref. 1683263, Wiener lab).
7. Homeostatic model assessment of insulin resistance (HOMA-IR): estimated using HOMA with the following formula: HOMA-IR = fasting insulin (µIU/ml)×fasting glucose (mg/dl)/405, where ELISA methodology was used to determining fasting insulin (Insulin ELISA Ref. EIA2935 DRG International, Inc.)

### Genotyping of the 45 T>G polymorphism of the *ADIPOQ* gene

***Genomic DNA extraction.*** A total of 5 ml peripheral blood (with EDTA as an anticoagulant) was obtained by venipuncture. For DNA extraction, the combined DNA-Salting out extraction technique was used, as described previously ^23^, of the Molecular Genetics laboratory of the Medical Genetics Unit of the Faculty of Medicine was used.

**Figure 1.**
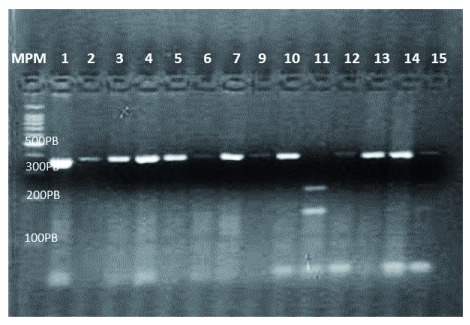
The 372 bp DNA fragment covering the region of interest amplified by PCR. The region of interest was amplified using this set of oligonucleotide primers: F5'-GAATGAGACTCTGCTGAGATGG-3' and R5'- TATCATGTGAGGAGTGCTTGGATG-3’.

***PCR amplification of 45 T>G polymorphism of the ADIPOQ gene.*** A 372 bp DNA fragment covering the region of interest was amplified by PCR, using this set of oligonucleotide primers: F5'-GAATGAGACTCTGCTGAGATGG-3' and R5'- TATCATGTGAGGAGTGCTTGGATG-3’ to amplify the region of interest ( Figure 1). The amplification conditions were the following: 95°C for 6 min, followed by 35 cycles of 95°C for 1 min, 45 sec at 56°C and 45 sec at 72°C and a final extension at 72°C for 5 min ^24^. For the amplification reactions, taq DNA polymerase from Promega was used.

***RFLP for the detection of the 45 T>G polymorphism of the ADIPOQ gene.*** The amplified fragment of 372bp was subjected to restriction analysis with the *Sma*I enzyme. A non-digested fragment (molecular weight, 372 bp) corresponded to the homozygous T/T genotype. The homozygous G/G genotype was evidenced by the presence of two fragments, one 219 bp and the other 153 bp. The product of the digestion of the heterozygous T/G genotype was evidenced by the presence of three fragments (372 bp, 219 bp and 153 bp), as shown in Figure 1.

### Statistical analysis

The statistical package SPSS for Windows, version 21.0 was used. Allelic and genotypic frequencies were calculated for the total population evaluated and for the MS-DM2 and control groups. The χ ^2^ test was used to compare frequencies and observed and expected values to evaluate the Hardy-Weinberg equilibrium (HWE). Means and medians were determined for biochemical, clinical and anthropometric characteristics accordingly. The normal distribution of the variables was verified by the Kolmorgov-Smirnov test. The comparison of the parametric variables with normal distribution was performed using Student's t-test, when 2 groups were compared and the Mann and Whitney U-test in those variables that had a non-normal distribution. A p-value <0.05 was considered statistically significant. Likewise, a binary logistic regression was carried out in order to obtain the odd ratio adjusted to a confidence interval of 95%, to estimate the relative risk of the variable under study. A p-value <0.05 was considered statistically significant.

## Results and discussion

### Study population characteristics

In this study, the +45 T>G polymorphism of the *ADIPOQ* gene was associated with the development of MS and DM2 in adult individuals from Maracaibo City. No prior information was found on the association of that polymorphism of the *ADIPOQ* gene with individuals diagnosed with MS and DM2. Complete raw data are available on OSF ^25^.

Clinical, biochemical and anthropometric characteristics of both groups (MS +DM2 and control patients) are shown in Table 2. Statistically significant differences were found when comparing the means and medians in almost all studied characteristics, with the exception of height and usCRP. Most of the parameters of MS and DM2 individuals were found to be higher than in healthy controls, with exception of HDL-c levels. In line with the findings of the present study, DM2 patients in Brazil, India and Russia ^18, 26, 27^ showed similar results in clinical characteristics, such as BMI, HOMA-IR, fasting glucose, TC, TG, HDL-c, LDL-c, systolic blood pressure (SBP) and diastolic blood pressure (DBP). Moreover, Sahli *et al*. ^28^ reported statistically significant differences in BMI, fasting glucose, TC, TG, HDL-c, LDL-c, DBP, and SBP in a Tunisian population. However, a DM2 Chinese Han population ^29^, showed no significant difference when comparing height and usCRP in diabetic and healthy control groups. These differences could be related to the heterogeneity of the populations, due to the fact that they may have totally different lifestyles, including feeding, physical activity, environmental factors, and customs, among others.

**Table 2.** Clinical and anthropometric characteristics of the subjects.

Clinical characteristics	All	MS+DM2 (n=44)	Control group (n=46)	p value
Gender				
Female, n	35	18	17	-
Male, n	55	28	27	-
Age (years) *	37 (17)	54 (9)	21.5 (3.5)	0.000 †
Height (m)	1.65 (0.08)	1.6 (0.09)	1.6 (0.06)	0.999 †
BMI (kg/m ^2^)	25.9 (4.8)	31.5 (2.8)	21.9 (1.4)	0.000 †
WC (cm)	89.50 (15.0)	106.50 (7.5)	77.00 (3.0)	0.000 †
CRP	0.54 (0.4)	0.712 (0.5)	0.364 (0.3)	0.47 ‡
Fasting Insulin (µUI/l) *	13.7 (4.6)	17.2 (4.5)	10.5 (2.9)	0.000 †
HOMA-IR *	2.3 (0.9)	3.1 (1.05)	1.5 (0.4)	0.000 †
FG (mg/dl) *	130.5 (39)	166.7 (44)	89.5 (6.5)	0.000 †
TC (mg/dl)	183.5 (29)	212.5 (47)	162 (20.2)	0.000 †
TG (mg/dl) *	108.45 (79)	213 (68.5)	59 (17.7)	0.000 †
HDLc (mg/dl) *	45 (8)	38.5 (6)	53 (8.7)	0.000 †
LDLc (mg/dl) *	102.6 (23.5)	131.3 (37.6)	93.1 (19.6)	0.000 †
SBP (mmHg)	120 (10)	130 (15)	110.0 (4.2)	0.000 ‡
DBP (mmHg) *	75 (7.5)	80 (9)	70.0 (6.7)	0.000 †

Values expressed as Median (interquartile range) unless indicated. *Variable expressed as logarithm. †Student’s t-test for independent samples. ‡Mann Whitney U-test. BMI, body mass index; WC, waist circumference; CRP, C-Reactive Protein; HOMA-IR, homeostatic model to estimate insulin resistance; FG, fasting glucose; TC, total cholesterol; TG, triacylglycerides; HDL-c, high-density lipoprotein cholesterol; LDL-c, low-density lipoprotein cholesterol; SBP, systolic blood pressure; DBP, diastolic blood pressure.

### Frequency of *ADIPOQ* genotypes

Table 3 depicts the allelic and genotypic frequencies of the +45T>G polymorphism of the *ADIPOQ* gene, in patients with MS-DM2 and healthy control patients. The T/T genotype was found in greater frequency in the total population, although patients with MS-DM2 showed a greater frequency, as described by Sahli *et al*. ^28^ in a Tunisian MS population. T/G and G/G genotypes were found in both groups but showed lower frequencies. The healthy controls showed a genotypic frequency of 27% and 5% for the T/G and G/G genotypes, respectively, which indicates that the homozygous T genotype is three times higher in healthy individuals. Other studies generated opposite results ^26, 27, 30, 31^, since the T/T genotype was found more frequently in healthy control patients than in DM2 ones.

**Table 3.** Allelic and genotypic frequencies of the +45T>G polymorphism of the *ADIPOQ* gene in control patients and patients with MS and DM2.

Genotype	Control Population		MS+DM2		Total population	
	n	Frequency (%)	N	Frequency (%)	n	Frequency (%)
T/T	30	68.0	41	89.0	71	79
T/G	12	27.0	4	9.0	16	18
G/G	2	5.0	1	2.0	3	3
Total	44	100	46	100	90	100
**Allele**	**n**	**Frequency**	**N**	**Frequency**	**n**	**Frequency**
T	72	0.82	86	0.93	158	0.88
G	16	0.18	6	0.07	22	0.12
Total	88	1.00	92	1.00	180	1.00

MS, metabolic syndrome; DM2, type 2 diabetes mellitus.

### Allelic frequencies

Regarding the alleles, a higher frequency of the T allele was observed over the G allele in both study groups, the T allele being more frequent in the population with MS-DM2, where the Hardy-Weinberg balance χ ^2^ = 5.99 is met, because the observed allele frequencies are as expected. Such findings relate to the MS population studied in Tunisia ^28^ but differ with the type 2 diabetes mellitus individuals studied in Brazil, India and Ireland ^26, 27, 31^ since the highest frequency of the T allele occurred in the healthy control group Table 4.

**Table 4.** Allelic and genotypic frequencies in various populations.

Population	Genotype (%)			Alleles		Study location (ref.)
	T/T	T/G	G/G	T	G	
DM2 individuals	71.4%	22.9%	5.7%	0.83	0.17	Ireland ^31^
Healthy controls individuals	84.8%	15.2%	0	0.92	0.8	
DM2 individuals	42.1%	36%	21.9%	-	-	Iran ^30^
Healthy controls individuals	53.5%	36.2%	10.2%	-	-	
MS individuals	88.5%	11.5%	0	0.94	0.6	Tunisia ^28^
Healthy controls individuals	83.6%	14.8%	1.6%	0.91	0.9	
DM2 individuals	64.7%	24.8%	10.4%	0.74	0.26	India ^27^
Healthy controls individuals	72.1%	22.4%	5.3%	0.79	0.21	
DM2 individuals	46.5%	47.5%	6.0%	0.70	0.30	Brazil ^26^
Healthy controls individuals	50.0%	42.5%	7.0%	0.71	0.29	

MS, metabolic syndrome; DM2, type 2 diabetes mellitus.

### Comparison of populations

As shown in Table 4, the population that differ the most from the one characterized in this study, turned out to be the Brazilian one ^26^, although Brazil is near Venezuela, the studied subjects were of Japanese ancestry; this could be the reason of such genetic differences although the lifestyles and customs are similar in both countries.

### Association of anthropomorphic characteristics with *ADIPOQ* status

Table 5 shows that there was a significant difference among waist circumference, fasting glucose, total cholesterol, triacylglycerides, LDL-c, DBP and SBP, for the T/T and T/G + G/G genotypes regarding the total population. Those that had the presence of the G allele of the polymorphism exhibited lower values except for the usCRP in which those subjects that carried the T allele had lower levels in this parameter.

**Table 5.** Association of the +45 T>G polymorphism of the *ADIPOQ* gene, according to MS-DM2 condition, with anthropometric, biochemical and clinical variables in the studied subjects.

Characteristics	Control subjects		p value	MS-DM2		p value	All		p-value
	T/T (n=30)	T/G+G/G (n=14)		T/T (n=41)	T/G+G/G (n=5)		T/T (n=71)	T/G+G/G (n=19)	
Gender									
Female, n	10	7	-	17	1	-	27	8	**-**
Male, n	20	7	-	24	4	-	44	11	**-**
Age (years)	21.5 (3.50)	21.00 (2.50)	*0.338 †	55.00 (9.00)	45.00 (2.00)	0.427 †	44.00 (18.50)	24.00 (10.00)	**0.002 †**
Height (m)	1.65 (0.05)	1.60 (0.06)	0.283 †	1.66 (0.11)	1.65 (0.02)	0.924 †	1.66 (0.09)	1.62 (0.06)	0.397 **†**
BMI	22.2 (1.52)	21.21 (1.75)	0.468 †	31.39 (3.08)	33.92 (1.85)	0.145 †	26.61 (4.37)	22.47 (4.38)	0.113 **†**
WC (cm)	77.50 (2.5)	76.0 (5.0)	0.519 †	106.00 (7.0)	116.00 (8.5)	0.523 †	96.50 (14.3)	79.00 (10.5)	**0.042 †**
CRP	0.364 (0.32)	0.356 (0.45)	0.315 †	0.776 (0.57)	0.670 (0.05)	0.365 †	0.541 (0.49)	0.575 (0.30)	0.938 **†**
Fasting insulin (µUI/L)	9 (2.15)	11.60 (3.90)	*0.441 †	17.00 (3.80)	24.30 (4.75)	**0.038 †**	13.70 (4.85)	13.50 (4.23)	0.718 **†**
HOMA-IR	1.3 (0.40)	1.70 (0.60)	0.848 †	3.10 (0.90)	4.40 (1.25)	*0.291 †	2.40 (0.90)	2.15 (0.70)	*0.818 **†**
Fasting glucose (mg/dl)	89.5 (6.50)	90.00 (7.05)	0.225 †	168.00 (43.00)	149.00 (29.70)	*0.374 †	138.00 (41.00)	102.00 (21.75)	*0.007 †
TC (mg/dl)	164 (22.00)	157.00 (20.50)	0.546 †	222.00 (45.50)	177.00 (21.50)	0.070 †	192.00 (36.00)	162.00 (21.50)	**0.001 †**
TG (mg/dl)	60.36 (18.50)	56.50 (17.21)	0.510 †	217.00 (59.50)	164.00 (80.50)	*0.967 †	127.00 (83.00)	77.00 (29.00)	*0.038 †
HDL-c (mg/dl)	53 (10.00)	52.00 (5.00)	0.588 †	39.00 (6.50)	32.00 (1.50)	0.174 †	44.00 (8.00)	50.00 (6.00)	0.566 **†**
LDL-c (mg/dl)	93.8 (21.50)	91.87 (12.00)	0.863 †	138.54 (39.45)	109.32 (22.94)	0.180 †	106.30 (32.40)	94.40 (13.70)	**0.003 †**
SBP (mmHg)	110 (7.50)	110.00 (5.00)	0.060 ‡	130.00 (15.00)	130.00 (7.50)	0.864 ‡	120.00 (13.50)	110.00 (3.50)	**0.007 ‡**
DBP (mmHg)	70 (7.50)	67.50 (5.00)	0.006 †	80.00 (9.00)	90.00 (10.00)	0.780 †	80.00 (10.00)	70.00 (5.00)	*0.004 †

Values expressed as median (interquartile range) unless indicated. *Variable logarithmically transformed for its normalization. †Student’s t-test for independent samples; ‡Mann-Whitney U-test. BMI, body mass index; CRP, C-reactive protein, HOMA-IR, homeostatic model to estimate insulin resistance, HDL-c, high-density cholesterol lipoproteins; LDL-c, low-density cholesterol lipoproteins; SBP, systolic blood pressure; DBP, diastolic blood pressure.

On the other hand, the control population (without MS-DM2) showed statistically significant difference regarding the DBP. In the MS-DM2, a significant difference was only observed in fasting insulin levels between groups. In addition, it was demonstrated that in these groups, the subjects who presented the G allele showed lower mean values than of those without this allele (homozygous T), except for the usCRP levels, which were higher in individuals with the G allele. Potapov *et al*. ^18^ obtained contradictory results in their study in a DM2 Russian population, since the fasting glucose levels showed higher values for subjects carrying the G allele, and in control subjects, a statistically significant difference was only found in SBP, which was higher in those individuals who carried the G allele.

### Previous studies

Several studies have reported the behavior of several anthropometric, clinical and biochemical characteristics in their total populations concerning the +45 T>G polymorphism, finding opposite data that the one found during this study. In a non-diabetic female population in Greece ^16^, fasting insulin and HOMA-IR levels were significantly lower in carriers of the rare +45G allele (T/G+G/G), which was also evidenced in Italian individuals with or without DM2 ^32^, in non-diabetic individuals in Taiwan ^33^, there were only significant differences in the BMI, with higher values for T allele carriers, unlike Punjab individuals ^34^ who those that carried the G allele displayed higher BMI and WC values.

According to Menzaghi *et al*. ^35^, the biology of these associations with anthropometric, clinical and biochemical characteristics is still unclear, for example, it is mentioned in their research that studies in animal models show that adiponectin is a potent insulin activator and modulator, in addition to regulating energy homeostasis and glucose tolerance. This relationship has also been found between the adiponectin levels in rodents and humans, which suggests that the +45 T>G *ADIPOQ* polymorphism can exert its action by diminishing or enhancing the expression of adiponectin, which can increase or decrease body weight and insulin resistance. In other populations, this variant may be associated with DM2, obesity and MS, whereas in other association studies, insulin resistance and DM2 are a consequence of the presence of the T allele.

Additionally, a binary logistic regression model was used to determine the influence of the +45 T/G polymorphism of the *ADIPOQ* gene on MS-DM2, where the odds ratio showed no association of this polymorphism as a risk factor predisposing to MS-DM2. The presence of the rare allele is considered a protection factor ( Table 6). These findings oppose those previously reported by Stumvoll *et al.* ^36^ who studied non-diabetic Germans and recognized that the +45G allele was associated with higher IR indexes. This led to lower insulin sensitivity compared to T allele carriers while those subjects who carried the polymorphism could present a higher risk of obesity. The same phenomenon was described in diabetics and non-diabetics in Northern India ^34^; however, no association between +45 T/G polymorphism and the development of DM2 was found in Koreans individuals with and without DM2 ^37^. The G allele of the +45 T>G polymorphism of the *ADIPOQ* gene has been associated with endocrine-metabolic pathology in Chinese individuals with and without MS in a similar population, whereas in Tunisia no association of polymorphism with MS was found ^28, 38^.

**Table 6.** Odds ratio (OR) of the variables related to metabolic syndrome (MS) and type 2 diabetes mellitus (DM2).

Variables	OR	p value
MS+DM2	0.261 (0.085–0.085)	0.019
DM2	0.319 (0.109–0.935)	0.037
MS	0.261 (0.085–0.805	0.019

Although there is a lack of consistency among studies, there is evidence that the 45G allele of the *ADIPOQ* gene plays a protective role in MS and DM2, whereas the 45T allele behaves as a risk allele for the development of obesity and insulin resistance, which are both related to the development of MS and DM2 ^32, 33, 39^, consistent with the findings in this study.

The +45 T>G polymorphism of the *ADIPOQ* gene is a silent mutation without its own biological effect, since it is a GGT to GGG substitution, which translates to Gly15Gly. Adiponectin’s role in insulin sensitivity and BMI, as well as its role in both MS and DM2 pathologies are associated with genetic alterations linked to the polymorphism-related disequilibrium. mRNA G allele transcripts vary, proving higher than those of T allele of heterozygous individuals. This suggests that promoter gene polymorphisms or other adiponectin gene regulatory elements could be involved in the decrease or increase of the expression of the studied adipokine, being able to affect the mRNA allowing the stability of the splice sites in the same, affecting the amount of circulating proteins ^28, 30^. It has also been observed that haplotypes that are in ligation unbalance with one or more functional variants can affect the levels of adiponectin in plasma, producing for example a change in the secondary structure of DNA, having a major influence on transcription, processing or translation; however, this is hypothetical, since the exact genetic mechanisms responsible for the expression of the specific allele have not been elucidated yet ^32^.

## Conclusions

In this study, biochemical, clinical and anthropometric characteristics corresponding to MS-DM2 were determined, and statistically significant differences were found between the means and medians of all the variables evaluated, except for height and usCRP. The genotype frequency of the +45 T/G polymorphism of the *ADIPOQ* gene, in the total population evaluated, was 79% for the homozygous T/T variant, 18% for the heterozygous T/G variant and 3% for the homozygous G/G variant. The allelic frequencies of the +45 T/G polymorphism were 0.88 for the T allele, while only 0.12 of the evaluated individuals exhibited the G allele.

Likewise, it was found that there is a statistically significant difference between the means and medians of WC, fasting glucose, total cholesterol, triacylglycerides, LDL-c, DBP and SBP, between genotypes T/T and T/G with respect to the total population. In addition, the total population with the G allele showed lower mean values for these variables than those without this allele (homozygous T), except for the values of CRP and HDL-c, which were higher in individuals with the G allele. Overall, an association was found between the +45 T>G polymorphism of the *ADIPOQ* gene with MS-DM2. The results suggest that the presence of the G allele exerts a protective effect on carrier individuals, thus preventing the onset of MS and DM2.

## Data availability

Open Science Framework: Association between +45T>G adiponectin polymorphism gene and type 2 diabetes mellitus and metabolic syndrome in a Venezuelan Population. https://doi.org/10.17605/OSF.IO/3HE6S ^25^.

The project contains the following extended data:

- Data Articulo.v3.xlsx (complete raw anthropometric data and *ADIPOQ* status).

Data are available under the terms of the Creative Commons Zero "No rights reserved" data waiver (CC0 1.0 Public domain dedication).
