# Supplementary material for: Association between +45T>G adiponectin polymorphism gene and type 2 diabetes mellitus and metabolic syndrome in a Venezuelan population
Source: F1000Res. 2019 Mar 14;8:292. [Version 1] doi: 10.12688/f1000research.16890.1 (PMC6530605; doi:10.12688/f1000research.16890.1)
Supplement: Peer review history

# Peer review: Reviewer response for version 1

**DOI:** 10.5256/f1000research.18466.r47149

**Published:** 2019-05-21

**Authors:** Tayebeh Pourmotabbed

**Affiliations:** 1 Department of Microbiology, Immunology and Biochemistry, University of Tennessee Health Science Center (UTHSC), Memphis, TN, USA

Adiponectin is a secretory protein and the most plentiful adipokine in circulation. It induces insulin sensitivity, has anti-inflammation and anti-atherogenic effects. Its low concentration is associated with an increase in body fat mass, insulin resistance and dyslipidemia. Adiponectin gene polymorphism has been shown to affect protein production and thus, its plasma concentration. In this manuscript, the authors have studied association between +45T>G polymorphism of the adiponectin gene (ADIPOQ) and risk of type 2 diabetes (DM2) and metabolic syndrome (MS) in subjects from Maracaibo municipality, Zulia state in Venezuela. Their data suggest that presence of the G allele at this position of adiponectin gene may protect the carrier individuals against MS and DM2, at least in this population.   

 The study is well thought of and well written, worth indexing. However, since low adiponectin level is associated with DM2 and MS and adiponectin gene polymorphism affects protein production; **in the case of sample availability and technical support**, it would be interesting to measure the level of adiponectin in the studied population. In addition, the presence of G allele may have an effect on stability of adiponectin RNA and thus the protein level, thus, determining the level of adiponectin RNA by semiquantitative PCR may shed some light on protective role of G allele.

I have read this submission. I believe that I have an appropriate level of expertise to confirm that it is of an acceptable scientific standard.

# Peer review: Reviewer response for version 1

**DOI:** 10.5256/f1000research.18466.r47649

**Published:** 2019-05-08

**Authors:** Na Zhu

**Affiliations:** 1 Columbia University, NYC, USA

Major concern:

1. In table 2, there is a significant age difference between case and control, it should be a covariate.
2. The precision of the p-value is not precise, I suggest the author try other tools for the statistical test to get higher precision.
3. In table 3, the allele frequency of this polymorphism varied among populations. The allele frequency of G in controls matched with the Latino population frequency in gnomAD. The allele frequency of G in cases is lower than gnomAD Latino population frequency but higher than African and Finnish. So, the ancestry is very critical for such analysis. How did the author decide the ancestry of each sample?
4. Even the cases were completely Latino descents, as the cohort is small, the confidence interval of T’s AF is large, it could be possible to result in a higher frequency of T with p-value >0.001.
5. In table 3, no association was present in the genotypic association or allelic association using a chi-square test. They also showed in table 6.
6. In table 4, the frequency in Ireland case and controls cohort reversed with the author’s cohort, they also mentioned in the text, how did the author to explain it?

 

 Minor concern:

1. In the section “Frequency of ADIPOQ genotypes”, it said, “the homozygous T genotype is three times higher in healthy individuals”. How was it from? I saw three times heterozygous T/G, was it a typo? If yes, it was not significant at all.
2. In table 2, I guess the age show median and interquartile range, then * should not be here.
3. In table 4, there is a typo, G’s frequency 0.08 in healthy Ireland and Tunisia study.
4. Several typos happened in table 5, the symbol is not consistent between case and control such as p-value in age, HOMA-R, and SBP.
5. Table 5 has duplicated information as table 2, they may be able to optimize their presentation.

 

 Overall, I think the writing and the calculation are correct. However, the evidence they supplied cannot support their conclusions, such as no association between case and control and contrary finding among studies. +45T>G is a polymorphism, the presence of its frequency in case and control could occasionally happen for such a small cohort. It is not convincing to draw the conclusion that the phenotypic difference resulted from +45 T>G, this could be due to other common or rare variants.

I have read this submission. I believe that I have an appropriate level of expertise to confirm that it is of an acceptable scientific standard, however I have significant reservations, as outlined above.

# Peer review: Reviewer response for version 1

**DOI:** 10.5256/f1000research.18466.r47504

**Published:** 2019-05-07

**Authors:** Eman T. Mehanna

**Affiliations:** 1 Department of Biochemistry, Faculty of Pharmacy, Suez Canal University, Ismailia, Egypt

The authors studied the relation of +45T>G adiponectin gene SNP and the parameters of type 2 diabetes mellitus and metabolic syndrome a Venezuelan population. They found that the rare G allele of this polymorphism may be protective against most of those parameters.

 Comments:

1. The title should be modified to: (Association of +45T>G adiponectin gene polymorphism with type 2 diabetes mellitus and metabolic syndrome in a Venezuelan population).
2. The language of the manuscript should be revised. Sometimes, the authors repeat sentences. For example, in the statistical analysis, the sentence (A p-value <0.05 was considered statistically significant) was repeated twice.
3. I did not understand the purpose of Table 6. I think genotype and allele frequencies should have been compared in the study groups using chi square test to assess the relation of the polymorphism with the development of metabolic syndrome. 
4. In the legend of Figure 1, please define the genotype of each lane in the figure. Genotype TG is not clear at all due to the relatively faint bands.
5. The conclusion is very long. I suggest summarizing it and mentioning only the important findings of the study. 

I have read this submission. I believe that I have an appropriate level of expertise to confirm that it is of an acceptable scientific standard.
